# Deconstructing Mitochondrial Dysfunction in Alzheimer Disease

**DOI:** 10.1155/2013/162152

**Published:** 2013-06-11

**Authors:** Vega García-Escudero, Patricia Martín-Maestro, George Perry, Jesús Avila

**Affiliations:** ^1^Centro de Biología Molecular “Severo Ochoa” (CSIC-UAM), 28049 Madrid, Spain; ^2^Centro de Investigación Biomédica en Red de Enfermedades Neurodegenerativas (CIBERNED), 28031 Madrid, Spain; ^3^University of Texas at San Antonio, San Antonio, TX 78249, USA

## Abstract

There is mounting evidence showing that mitochondrial damage plays an important role in Alzheimer disease. Increased oxygen species generation and deficient mitochondrial dynamic balance have been suggested to be the reason as well as the consequence of Alzheimer-related pathology. Mitochondrial damage has been related to amyloid-beta or tau pathology or to the presence of specific presenilin-1 mutations. The contribution of these factors to mitochondrial dysfunction is reviewed in this paper. Due to the relevance of mitochondrial alterations in Alzheimer disease, recent works have suggested the therapeutic potential of mitochondrial-targeted antioxidant. On the other hand, autophagy has been demonstrated to play a fundamental role in Alzheimer-related protein stress, and increasing data shows that this pathway is altered in the disease. Moreover, mitochondrial alterations have been related to an insufficient clearance of dysfunctional mitochondria by autophagy. Consequently, different approaches for the removal of damaged mitochondria or to decrease the related oxidative stress in Alzheimer disease have been described. To understand the role of mitochondrial function in Alzheimer disease it is necessary to generate human cellular models which involve living neurons. We have summarized the novel protocols for the generation of neurons by reprogramming or direct transdifferentiation, which offer useful tools to achieve this result.

## 1. Introduction

Alzheimer disease (AD) is characterized by the presence of two aberrant structures in the brain of the patients, senile plaques and neurofibrillary tangles, together with marked neuronal death [1]. Senile plaques are filamentous aggregates of amyloid-beta peptide (Aβ) [2], whereas the main component of neurofibrillary tangles is the microtubule-associated protein tau [3]. This picture of the patients' brain showing plaques and tangles can be found at the end of the disease, usually at autopsy; however, using imaging techniques like positron emission tomography these aggregates can be detected *in vivo* in Alzheimer's patients by using compounds like the Pittsburg compound B for amyloid [4] or 18F-THK23 for Tau aggregates [5].

It is suggested that Alzheimer disease is a silent neurodegeneration where neuronal damage occurs before the diagnosis of the disease. Thus, plaques and tangles may appear before the onset of the disease symptoms [6]. On the other hand, mitochondrial dysfunction is one of the earliest and most prominent features in vulnerable neurons in the brain of AD patients [7] and likely in other neurodegenerative disorders [8].

There are at least two types of Alzheimer disease: familial Alzheimer disease (FAD) and sporadic Alzheimer disease (SAD). In FAD, the causes of the disease are the presence of specific mutations in at least one of the three genes identified as amyloid precursor protein (APP) and presenilin-1 and -2 (ps-1 and ps-2) [9]. However, little is known about the cause of the onset of SAD. It is known that the main risk for AD is aging related to oxidative damage [10]. It has been found that oxidative damage may facilitate the expression of beta-secretase (BACE1), a protein involved in the generation of Aβ [11, 12]. Moreover, it has been shown that oxidative stress induces a pathogenic PS1 conformational change in neurons *in vitro*, increasing Aβ42/40 ratio [13]. In FAD, increased Aβ may result in mitochondrial dysfunction and augmented ROS levels [14]. In SAD, the reverse has been suggested. Mitochondria-derived reactive oxygen species result in an enhanced amyloid-beta formation [15] and the Aβ increase may lead to further mitochondrial dysfunction, resulting in even higher ROS levels [16]. Then, the cycle repeats and degeneration increases. Also, oxidative stress may facilitate tau phosphorylation at some of the sites found to be modified in AD patients [17, 18]. On the other hand, tau accumulation causes mitochondrial distribution deficits in a mouse model for AD [19].

## 2. Mitochondrial Damage in AD

### 2.1. Oxidative Stress and Energy Production

**Figure 1:**
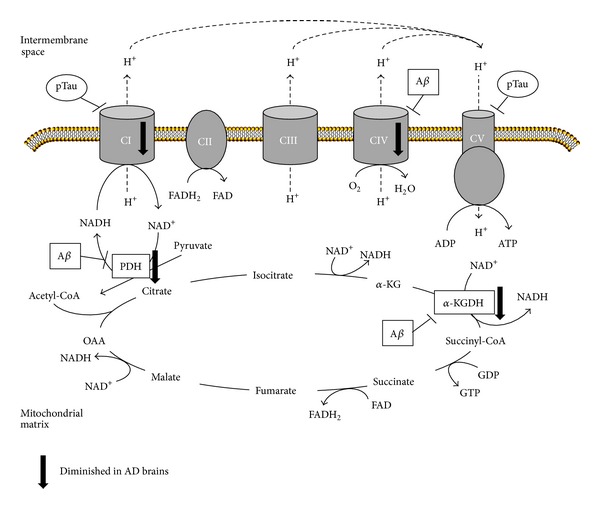
*AD-related alterations of mitochondrial respiratory chain and tricarboxylic acid cycle.* Scheme of alterations in protein levels found in Alzheimer disease brains as well as the targets of amyloid-β (Aβ) and phosphorylated Tau (pTau). Oxidative phosphorylation complexes are labeled as CI to CIV. PDH: pyruvate dehydrogenase, *α*-KG: *α*-ketoglutarate, *α*-KGDH: *α*-ketoglutarate dehydrogenase, OAA: oxaloacetate.

Oxidative stress is a primary event in the development of AD [10]. This oxidative stress may be due to the presence of dysfunctional mitochondria resulting in generation of reactive oxygen species [20]. Mitochondria generate cell energy as electrons flow through mitochondrial complex I to IV of the electron transport chain, from donors with lower redox potential to acceptors with higher redox potential. The final acceptor is oxygen that is reduced to water and the generated energy drives the phosphorylation of ADP to ATP by the mitochondrial complex V (or ATP-synthase) (Figure 1). Although the transport of the electrons through mitochondrial complexes is an efficient process, some reactive oxygen species (ROS) may be produced. Dysfunctional mitochondria generate high levels of ROS that may be toxic for cells with a long life span and a deficiency in antioxidant defenses, such as neurons [21]. Additionally, mitochondria are at the same time a target of ROS causing the oxidation of their components such as mtDNA, lipids, and proteins increasing mitochondrial deterioration. Mitochondrial dysfunction is one of the earliest and most prominent features of AD [22, 23]. In fact, a decreased expression of either nuclear or mitochondrial genes of the oxidative phosphorylation in the neocortex of AD patients has been shown to correlate with progressive reductions in brain glucose metabolism that can be visualized by positron emission tomography [24, 25]. Testing mitochondrial function in a triple transgenic mouse model for AD, a clear deregulation of oxidative phosphorylation proteins was found [26]. Deregulation of complex I was related to tau toxicity as found in other animal models [27] whereas deregulation of complex IV has been described to be Aβ dependent [28, 29] (Figure 1).

Alterations of several enzymes involved in the tricarboxylic acid cycle such as pyruvate dehydrogenase and *α*-ketoglutarate dehydrogenase have been reported in Alzheimer disease (Figure 1). Postmortem brains showed a reduction of pyruvate dehydrogenase, ATP-citrate lyase, and acetoacetyl-CoA thiolase correlating with decreased production of acetylcoenzyme A and the subsequent cholinergic defects observed in these patients [30]. Reduced activity of *α*-ketoglutarate dehydrogenase was also observed in brain tissue as well as in peripheral cells from AD patients [31, 32]. Additionally in AD brain, there is a loss of *α*-ketoglutarate-enriched cells, therefore causing the degeneration of *α*-ketoglutarate-enriched areas (cortical layers II and IV) which are the ones that are selectively degenerated in AD [33].

On the other hand, products of the toxic action of ROS, like hydroxynonenal (HNE), or the presence of quinones (like coenzyme Qo) may facilitate the self-assembly of Tau protein into fibrillar polymers similar to those paired helical filaments present in the brain of AD patients [34]. These findings suggest another possible link between oxidative stress, neuronal dysfunction, and AD.

It is possible that mitochondrial dysfunction in AD patients not only takes place in the central nervous system but also in cells from peripheral tissues. Increased oxidative stress levels and reduced antioxidant defenses have been observed in AD fibroblasts [35]. Regarding this, it has been described that lipoic acid and N-acetylcysteine may decrease the mitochondrial-related oxidative stress in Alzheimer disease patients [36].

### 2.2. Mitochondrial Dynamics Alteration

Mitochondria are highly dynamic organelles, ranging from giant tubular networks to small round entities through rapid and reversible fission and fusion processes. Mitochondria failure may arise from a deficient dynamic balance of mitochondrial fission and fusion that, in AD, is greatly shifted toward fission and it may result in the presence of dysfunctional mitochondria in damaged neurons [37].

**Figure 2:**
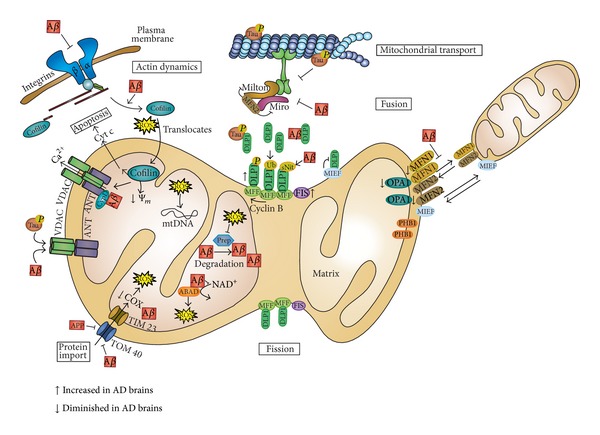
*Mitochondrial alterations found in AD.* Scheme of the effect of amyloid-β (Aβ) and phosphorylated-Tau (pTau) over mitochondrial dynamics, transport, protein import, membrane permeabilization, and apoptosis as well as actin dynamics. Alterations of the levels of involved proteins found in AD brains are also summarized. DLP1: dynamin-like protein 1, MFF: mitochondrial fission factor, FIS: fission 1, MIEF: mitochondrial elongation factor, MFN1: mitofusin 1, MFN2: mitofusin 2, OPA1: optic atrophy 1, PHB1: prohibitin 1, ABAD: Aβ-binding alcohol dehydrogenase, Prep: presequence protease, VDAC: voltage-dependent anion channel, ANT: adenine nucleotide translocase, CypD: cyclophilin D, Cyt C: cytochrome *c*, COX: cytochrome *c* oxidase, Ψ_*m*_: mitochondrial membrane potential, and mtDNA: mitochondrial DNA.

The delicate balance of fission and fusion is regulated by several mitochondrial proteins (Figure 2). Fission requires several outer mitochondrial membrane (OMM) proteins such as the GTPase dynamin-like protein 1 (DLP1, also known as dynamin-related protein, Drp1) that is recruited from the cytosol to the OMM for fission [38]. This process depends on its GTPase activity as well as on posttranslational modifications such as phosphorylation, S-nitrosylation, sumoylation, and ubiquitination [39–42]. Other OMM proteins involved in fission are Fis1, which plays a regulatory role, and mitochondrial fission factor, Mff, which is fundamental for the mitochondrial recruitment of DLP1 [43]. On the other hand, mitochondria fusion depends on other GTPases such as mitofusin 1 and 2, responsible for outer membrane fusion, and optic atrophy, Opa1, that carries out fusion of the inner membrane and is also important for cristae formation and mtDNA inheritance [44]. The involvement of mitochondrial elongation factor, MIEF1, in mitochondrial fusion in vertebrates has recently been described. This factor recruits and inactivates DLP1 executing a negative effect on fission and actively promotes fusion in a manner distinct from mitofusins [45].

Mitochondrial dynamics are critical for the maintenance of mitochondrial integrity and functions including energy metabolism, ROS generation, and apoptosis regulation [7]. Fusion permits the proper distribution of mitochondrial components such as lipids membranes, oxidative phosphorylation complexes, and mtDNA. Moreover, fusion is important in maintaining the proper mitochondrial ultrastructure and elongation is a mechanism for mitochondria to escape autophagy-mediated destruction. On the other hand, fission permits the recycling of irreversibly damaged mitochondria by mitophagy and plays an important role in the proper assembly of mitochondrial electron transport chain complexes. A proper balance of fusion and fission proteins is fundamental for the correct distribution of the mitochondria in the cell [44]. This is particularly important for neurons that may have very long axons and also for the function of synapses, which are subcellular regions with high metabolic requirement [46].

Possible dysfunction of mitochondrial dynamics proteins has been tested in models for neurodegenerative disorders [47, 48], Alzheimer disease being one of them [49]. In fact, abnormal mitochondrial dynamics and synaptic degeneration are considered early events in Alzheimer Disease (extensively discussed in Reddy et al. [50]). Mitochondrial dynamics alteration has been found either in neurons or fibroblasts in AD patients and models [7]. Mitochondria distribution has also been found altered in both cell types characterized by their accumulation into perinuclear areas. However, while neurons exhibit increased fragmentation, fibroblasts show elongated and highly interconnected mitochondrial network [47]. Vulnerable neurons in AD brain exhibit significant reduction in mitochondrial length and increased width with a significant increased overall size consistent with unopposed fission suggesting alterations of mitochondrial dynamics [48]. In agreement with these findings, an abnormal distribution of mitochondria was found in pyramidal neurons of AD-affected individuals where mitochondria were redistributed away from axons in the pyramidal neurons [51]. Accordingly, levels of fusion proteins OPA1, Mfn1, and Mfn2 were significantly reduced whereas levels of Fis1 were significantly increased in AD (Figure 2). In the case of the fission protein DLP1, while some authors have described a reduction in neurons [47, 52] and fibroblasts of sporadic patients [47], others have shown an increase [53]. These differences can be explained because the major pool of DLP1 is cytosolic and its recruitment on mitochondrial membrane to mediate fission events depends on posttranslational modifications [7] (Figure 2). In this sense, higher DLP1 levels in mitochondrial fraction [51] as well as increased Ser616 phosphorylation and S-nitrosylation in AD brains [40] have been described. Primary hippocampal neurons treated with Aβ-derived diffusible ligands (ADDLs) demonstrated shortened mitochondria in neurons and alteration of fission and fusion proteins [51]. Moreover, time-lapse recordings in these neurons showed impairment of both, fission and fusion processes, with fusion process being more severely affected [51]. Recent evidence has shown an abnormal interaction of Aβ monomers and oligomers with DLP1 that increases with the progression of the disease suggesting a possible cause of abnormal mitochondrial dynamics and synaptic damage [53]. On the other hand, the expression of AD-causing Swedish APP mutation in M17 cells also induced shorter and fatter mitochondria, with a slight but significant increase in size, but a decrease in the total mitochondrial number while the number of damaged mitochondria was increased [48]. Similar observations have been found in transgenic mice (Tg2574) harboring the APP Swedish mutation [54].

Abnormal mitochondrial morphology has been found in fibroblasts from sporadic AD patients, where they become significantly elongated and form a highly connected network [47]. This discrepancy in mitochondrial morphology may be due to differences in the expression pattern of proteins involved in dynamics, showing decreased DLP1 and unchanged OPA1. Similar differences between fibroblasts and neurons have been also found in Parkinson's disease [7].

Mitochondrial mobility has also been altered in AD causing a mitochondrial reduction in neurites [7] (Figure 2). Aβ induces a reduction in motile mitochondria [55] and ADDL impairs anterograde and retrograde axonal transport of mitochondria in hippocampal neurons [56]. Primary neurons from Tg2576 APP transgenic mice showed a specific impairment of anterograde mitochondrial transport. These results suggest that mitochondrial fission/fusion and mitochondrial transport can be coupled. In fact, it has been demonstrated that Mfn2 interacts with Miro and Milton, two adaptor proteins involved in the regulation of mitochondrial transport [57], although further work is necessary to clarify this relationship.

The alteration in mitochondrial dynamics leads to severe consequences in the cell such as structural changes in the cristae formation and assembly of electron transport complex compromising bioenergetics and causing calcium dyshomeostasis, increased oxidative stress, mitochondrial DNA damage, and synaptic dysfunction (reviewed in [7]).

## 3. Relationship between Mitochondrial Dysfunction and AD-Related Pathology

### 3.1. Amyloid Beta

In FAD, an increase in the level of Aβ may result in oxidative damage [22, 58, 59]. APP and Aβ accumulate in mitochondrial membranes causing structural and functional damage (reviewed in [60]). Nonglycosylated full-length and C-terminal truncated APP has been found to accumulate in the protein import channels of mitochondria of human AD brains [61] (Figure 2). APP forms stable complexes with the translocase of the outer mitochondrial membrane 40 (TOM40) import channel and the translocase of the inner mitochondrial membrane 23 (TIM23) inhibiting the entry of nuclear-encoded cytochrome *c* oxidase subunits IV and Vb proteins, which was associated with decreased cytochrome *c* oxidase activity and increased ROS production. Additionally, an interaction has been discovered between Aβ and phosphorylated Tau with voltage-dependent anion channel 1 (VDAC1) in the brains of AD patients and from APP, APP/PS1, and 3XTg AD mice which may block the mitochondrial pores leading to mitochondrial dysfunction [62].

Amyloid-beta interacts with the mitochondrial protein ABAD (Aβ-binding alcohol dehydrogenase) which is upregulated in the temporal lobe of AD patients as well as in AβPP transgenic mice [63] (Figure 2). This complex prevents the binding of nicotinamide adenine dinucleotide NAD+ to ABAD, thereby changing mitochondrial membrane permeability and reducing the activities of respiratory enzymes causing elevated ROS.

Moreover, it has been suggested that the oxidative stress induced by Aβ may oxidize and inactivate presequence protease, PreP, one of the proteins involved in Aβ degradation in the mitochondria thus increasing Aβ concentration in mitochondrial matrix and its pathologic effects [64] (Figure 2).

Amyloid-beta has been also involved in alterations of mitochondrial dynamics. In fact, the overexpression of human APP Swedish double mutation in neuroblastoma cell lines induces a higher percentage of highly fragmented and slower mitochondria correlating with an alteration of the levels of the proteins involved in mitochondrial dynamics such as increased fission protein Fis-1 and reduced levels of fusion proteins like OPA1 and DLP-1 [48]. In agreement with these *in vitro* findings, an abnormal distribution of mitochondria was also found in pyramidal neurons of AD-affected individuals.

Additionally, Aβ enhances nitrosative stress-inducing s-nitrosylation of DLP1, which favors mitochondrial fission followed by mitochondrial depletion from axons and dendrites and subsequently synaptic loss [40] (Figure 2).

Mitochondrial Aβ may also interact with cyclophilin D, an integral part of the mitochondrial permeability transition pore (mPTP), which potentiates free radical production, causes synaptic failure, and promotes the opening of the mPTP leading to apoptosis [65] (Figure 2).

On the other hand, Aβ accumulation could result in cytoskeletal aberrations [66] (Figure 2). Aβ oligomers interact with integrins leading to improper control of focal adhesion assembly and signaling, therefore causing the dysregulation of cofilin, which is involved in the regulation of actin dynamics. The inhibition of actin dynamics is associated with increased ROS production and reduced mitochondrial potential. Moreover, cofilin in response to oxidative stress translocates to the mitochondria where it induces swelling, a drop in mitochondrial membrane potential, and cytochrome *c* release promoting the opening of mPTP and apoptosis.

### 3.2. Presenilin

Presenilins 1 and 2 are multitransmembrane proteins that associate with nicastrin, APH-1, and PEN-2, form high-molecular *γ*-secretase complex, and are involved in Aβ production byintramembrane cleavage of APP. Ps-1 gene mutations are the most prevalent in FAD, but besides the generation of Aβ, little is known about its implication in mitochondrial dysfunction and oxidative damage. It has been demonstrated that presenilin-1 [67] and presenilin-2 are also located in mitochondria as part of the *γ*-secretase complex [68]. Noteworthy, presenilin mutations have been shown to sensitize cells to apoptosis by mechanisms suggested to involve impaired mitochondrial function and Ps-2/*γ*-secretase activity can modify mitochondrial membrane potential [69]. Moreover, ps-2 KO mouse embryonic fibroblasts exhibit lower basal respiratory rate. On the other hand, in at least two transgenic mouse models expressing human tau with AD mutations at presenilin-1, PS1M146L [70] and PS1A246E [71], the existence of mitochondrial abnormalities prior to cognitive deficits has been described. Also, in the case of PS1M146L mice, it was found that the mutation increases mitochondria ROS formation and oxidative damage. Finally, it has been recently shown that presenilins and *γ*-secretase activities are concentrated in a specialized subcompartment of the endoplasmic reticulum (ER) that is physically and biochemically connected to mitochondria, called mitochondria-associated ER membranes (MAM) which are involved in mitochondrial function and dynamics, among others [72, 73]. Either in presenilin KO mice or fibroblasts from FAD and SAD patients, MAM function is increased correlating with a significantly increased area of apposition between ER and mitochondria.

### 3.3. Tau

Tau is involved in the axonal transport of organelles such as mitochondria [74]. Hyperphosphorylated Tau may block the transport of mitochondria leading to energy deprivation and oxidative stress at the synapse as well as to neurodegeneration [75] (Figure 2). Analysis of brain proteins from P301L mutant human tau transgenic mice revealed deregulation of mitochondrial respiratory chain complex components such as complex V and reduced complex I activity as well as an impaired mitochondrial respiration with the subsequent ROS accumulation with aging [27] (Figure 1). Accordingly, the overexpression of P301L tau mutation in human neuroblastoma cells has been shown to induce substantial complex I deficit accompanied by decreased ATP levels and increased susceptibility to oxidative stress [76]. This was paralleled by pronounced changes in mitochondrial morphology, decreased fusion and fission rates accompanied by reduced expression of OPA-1 and DLP-1. In contrast, the overexpression of wt tau exhibited protective effects on mitochondrial function and dynamics including enhanced complex I activity. Moreover, an abnormal interaction of hyperphosphorylated Tau and mitochondrial fission protein DLP-1 has been described suggesting a relationship with mitochondrial dynamics alteration [77] (Figure 2). Other researchers have found that the expression of human tau mutations in both *Drosophila* (R406W) and mouse neurons (P301L) results in elongation of mitochondria, which is accompanied by mitochondrial dysfunction and cell cycle-mediated cell death [78]. We have previously mentioned an interaction of phospho-Tau and VDAC1 that may in turn block the mitochondrial pores leading to mitochondrial dysfunction [62] (Figure 2). On the other hand, increased oxidative stress has been shown to cause Tau hyperphosphorylation in a superoxide dismutase 2 knockout mouse model [79]. Furthermore, the inhibition of complex I with annonacin led to a redistribution of Tau from the axons to the cell body which correlates with a retrograde transport of mitochondria and finally to cell death [80]. Lastly, the downregulation of the proteins involved in the axonal transport of mitochondria such as Miro and Milton in *Drosophila* has shown loss of axonal mitochondria that promotes Tau phosphorylation in Ser262 via partitioning defective-1 (*Drosophila* homolog of mammalian microtubule affinity-regulating kinase) causing late-onset neurodegeneration in the fly [81].

## 4. The Use of Mitochondria-Targeted Antioxidants in AD

Since, as previously indicated, mitochondrial dysfunction and oxidative stress may play a role in the development of AD, many efforts have been proposed to demonstrate the therapeutic potential of antioxidants in this disease. Different components such as vitamin E [82], curcumin [83], *Gingo biloba* [84], and melatonin [85] have demonstrated their potential to reduce Aβ levels and improve mitochondrial function and cognitive behavior in animal models of AD. However, clinical trials using these antioxidants or others, such as huperzine A, have shown only modest or no effect in cognitive function. The modest effect may be related to unsuccessful cross of the blood-brain barrier, not well-thought-out experimental design of the clinical trials, or the late stage of patients involved [50].

To improve this poor result many efforts have been done to develop mitochondria-targeted antioxidants, such as triphenylphosphonium-based antioxidants (MitoQ, MitoVitE, Mito-*α*-lipoic acid, MitoPBN) [86], cell-permeable small peptide-based molecules (SS31, SS02, SS19, and SS20) [87], and choline esters of glutathione and N-acetyl-l-cysteine [88]. The first group results from the combination of lipophilic triphenylphosphonium cation with ubiquinol, *α*-tocopherol, *α*-lipoic acid, and *α*-phenyl-N-tert-butylnitrone, respectively. Due to their positive charge they are accumulated several hundredfold within mitochondria driven by the membrane potential, enhancing the protection of mitochondria from oxidative damage [86]. MitoQ accumulates in mitochondria driving the conversion of H_2_O_2_ to H_2_O and O_2_, reducing the toxic insult of free radicals.

Szeto-Schiller or SS peptides are a serial of small cell-permeable antioxidant peptides which have a sequence motif that allows them to target mitochondria [87]. They scavenge H_2_O_2_ and ONOO– and inhibit lipid peroxidation. Their antioxidant activity is attributed to the tyrosine or dimethyltyrosine (Dmt) residue. Dmt has demonstrated to be more effective than tyrosine in scavenging of ROS. SS31 (H-D-Arg-Dmt-Lys-Phe-NH2) has demonstrated its efficacy in rodent models of different diseases.

The antioxidant effect of MitoQ and SS31 has been tested *in vitro* in mouse cell models of AD [89]. Both components were able to prevent the effects of Aβ in mouse neuroblastoma (N2a) cells, such as increased expression of mitochondrial fission genes, decreased expression of fusion genes, peroxiredoxins, and endogenous cytoprotective antioxidant enzymes, and, increased number of intact mitochondria and neurite outgrowth. Additionally, in neurons from a mouse model of AD (Aβ precursor protein transgenic mouse, Tg2576 line) incubated with Aβ, MitoQ and SS31 achieved an increase in neurite outgrowth and a decrease in cyclophilin D expression. Posterior work using primary neurons from Tg2576 mice [54] confirmed the capacity of SS31 to mitigate the effects of oligomeric Aβ, such as decreased anterograde mitochondrial movement, increased mitochondrial fission, decreased fusion and structurally damaged mitochondria, abnormal mitochondrial and synaptic proteins, defective mitochondrial function, and apoptotic neuronal death. SS31 was able to restore mitochondrial transport and synaptic viability and decreased the percentage of defective mitochondria, demonstrating its protective effect from Aβ toxicity.

Moreover, MitoQ has been shown to prevent cognitive decline in 3xTg-AD mice as well as early neuropathology, such as oxidative stress, Aβ accumulation, astrogliosis, synaptic loss, and caspase activation [90].

Another example of mitochondria-targeted antioxidants could be the compound SKQ1 (plastoquinonyl decyltriphenylphosphonium), a membrane-penetrating cation that is specifically accumulated in the inner mitochondrial membrane [91]. SKQ1 lowers the rate of ROS formation in the respiratory chain due to mild uncoupling. SKQ1 reversed the appearance of a typical behavioral trait of aging in rats [92].

Further evidence suggesting the therapeutic capacity of mitochondria-targeted antioxidants was recently obtained from the *in vivo* studies using APP transgenic mice that carried the human mitochondria-targeted catalase (MCAT) gene [93]. These mice lived 5 months longer than did APP mice. Their brain sections showed a reduction in the levels of full-length APP, C-terminal fragment 99, BACE1, Aβ levels (40 and 42), Aβ deposits, and oxidative DNA damage relative to the brain sections from the APP mice. Additionally, significant increased levels of soluble APP*α* and C-terminal fragment 83 were found in the MCAT/APP mice, suggesting that oxidative stress plays a primary role in AD etiopathology.

All these findings indicate that mitochondria-targeted molecules may be an effective therapeutic approach to treat patients with AD.

## 5. Removal of Defective Mitochondria

Autophagy is a normal cellular recycling process that involves degradation of intracellular components including proteins, protein complexes, and organelles through lysosomal degradation. Mitochondrial function is regulated by autophagy in a process named mitophagy, in which dysfunctional mitochondria are recycled by engulfment into autophagosomes that then fuse with lysosomes for their content degradation. The segregation of damaged mitochondria depends on fission and fusion events that, as we have previously discussed, are altered in AD. Although increased mitochondrial autophagy in AD has been described [94], further studies will be necessary to clarify if this is a protective process because they may not be properly recycled by fusion with lysosomes or it may not be selective for damaged mitochondria.

One of the mechanisms described for the regulation of mitochondrial recycling by autophagy involves the E3 ubiquitin ligase Parkin. After mitochondrial damage, PTEN-induced kinase 1 (PINK1) is stabilized in mitochondria inducing the recruitment of Parkin [95]. Parkin-mediated ubiquitination recruits autophagy adapter proteins, such as p62, which interacts with LC3 mediating the cargo engulfment into autophagosomes. Parkin ubiquitinates several mitochondrial proteins, such as VDAC1 [96] and mitofusins that may be involved in the segregation of damaged mitochondria via an inhibition of mitochondrial fusion events [97]. Therefore the alterations described in mitochondrial dynamics-related proteins and autophagy in AD may affect the mitophagy increasing mitochondrial damage and ROS accumulation.

In AD, Aβ, and Tau aggregation has been associated with mitochondrial damage, oxidative stress, and cytoskeletal alteration of neurons. Autophagy plays a fundamental role in neuronal function and is intensively involved in AD-related protein aggregation [98]. Indeed, it has been demonstrated that autophagy is the major degradational pathway following unfolded protein response activation in neuronal cells, an early event in AD brain, suggesting a connection between its activation and the observed autophagic pathology [99]. Accordingly, an accumulation of autophagic vesicles in the cortex of AD patients compared to nondemented ones has been shown [98]. Moreover, an increase of autophagic vesicles containing mitochondria in pyramidal neurons from AD patients has been found, suggesting a mitophagy alteration [94, 100]. According to this, Parkin, one of the proteins involved in the target of mitochondria to be degraded by mitophagy, has been shown to be reduced in the cortex of AD brains [101]. Additionally, autophagy alterations have been described in AD brain and animal models. Beclin 1, a protein that plays a key role in autophagy, has been shown to be diminished in the affected brain regions in AD patients early in the disease process [102]. In the same work, in an APP transgenic mouse model, the downregulation or overexpression of beclin1 increased or diminished, respectively, the Aβ accumulation, extracellular Aβ deposition, and neurodegeneration, highlighting the relevance of autophagy in AD-related pathology. Moreover, a link between FAD and autophagy has been recently indicated, showing that autophagy requires functional Ps-1 for lysosomal maturation and that is impaired by Alzheimer-related ps-1 mutations [103]. Thus, ps-1 mutations could indirectly affect mitochondrial function by impairing its recycling by mitophagy.

On the other hand, autophagy has been proposed to play an active role in AD pathogenesis. In this regard, autophagic vesicles have been demonstrated to be an active compartment for Aβ generation and their abnormal accumulation in affected neurons of the AD brain contributes to Aβ deposition [104].

Due to the crucial role of autophagy in AD, the Moussa group proposed the induction of autophagy by overexpression of Parkin as a therapeutic strategy. Parkin could ubiquitinate and decrease intracellular Aβ levels and plaque deposition by a synergistic activation of proteasomal degradation [105] and Beclin1-dependent autophagic clearance [106]. Additionally, Parkin-induced autophagy facilitated clearance of vesicles containing debris and defective mitochondria counteracting oxidative stress and preventing mitochondrial dysfunction [106]. Parkin reverses intracellular Aβ accumulation and its negative effects on proteasome function [101].

Other strategies for the induction of autophagy as a therapeutic strategy in AD have been tested in animal models for the disease. With this aim several molecules have been tested such as rapamycin [107, 108], cystatin B [109], trehalose [110], *scyllo*-Inositol [111], and latrepirdine [112], although effects on improving mitochondrial recycling were not studied in these works.

## 6. Novel Models for the Study of AD

Hitherto, the majority of observations about mitochondrial failure come from the study of animal models of familial AD, patient-derived nonneuronal cells, and postmortem analysis of the patient's brain. As we have previously discussed, mitochondria are highly dynamic organelles which coordinate a vast amount of cellular functions; therefore, although the analysis of the brain can give us clues about alterations in the amount of involved proteins and in the structural changes by image analysis, for the proper study of mitochondrial function the use of living cells is necessary. In this direction, the use of patient-derived fibroblasts can be very helpful but we should not forget that these are not the cells that degenerate in the disease, so they might be subject to some kind of compensatory mechanism, making them very different from neurons. Additionally, fibroblast environment, energy requirements, and protein expression pattern and morphology are very different from those of neurons, so the extrapolation of the results obtained in this kind of cells should be taken cautiously. FAD animal models or the expression of AD human mutations in human neuroprecursor cells offers us the possibility of studying mitochondrial function in a cellular model related to the disease. However, we should take into consideration that the majority of AD cases are sporadic or are not related to any known mutation associated with the disease. For these reasons, there is an increasing effort in generating neuronal cells derived from SAD patients.

A revolutionary work in 2006 by Takahashi and Yamanaka demonstrated for the first time that adult differentiated cells such as fibroblasts are able to be retrodifferentiated to generate stem cells [113]. Four transcriptions factors, Oct3/4, Sox2, c-Myc, and Klf4, were enough to induce pluripotency in primary fibroblast indicating that cell programming could be reversed. This innovative concept opens the possibility of generating neuronal cells from patient-derived fibroblasts having a deep impact on the study of neurodegenerative diseases. Noteworthy, Yamanaka obtained the Nobel Prize in 2012 in recognition to his discovery. After this pioneer work many efforts have been done to successfully reproduce this result in human fibroblasts [114] as well as optimize the protocol by changing the transcriptional factors [115], as well as substituting them by small molecules [116–118] or miRNAs [119–121] for transcription factors.

An increasing number of papers have come out in the last few years using fibroblasts for the generation of induced pluripotent stem cells (iPS), some of which are focused in the generation of neurons from these iPS for the study of Alzheimer disease [122, 123]. Using this approach of generating neurons from presenilin-associated FAD patient's fibroblasts, increased Aβ42 production and secretion was found [122]. Similar studies using sporadic and APP duplication associated AD fibroblasts have also found an increase in Aβ40, phospho-Tau (Thr 231), or in active GSK3 (lacking Ser9 phosphorylation) as well as the accumulation of early endosomes [123].

More recently, direct conversion of fibroblast to functional neurons (induced neurons, iN) by just three transcription factors, Ascl1, Brn2 (also called Pou3f2), and Myt1l has been described [124]. This was subsequently reproduced by using human fibroblasts [125]. Also, microRNA-mediated conversion of human fibroblasts to neurons has been indicated, although its efficiency is increased by the addition of transcription factors [126]. Additionally, a combination of one microRNA and two transcription factors appears to be sufficient to reprogram human fibroblasts to functional neurons [127]. Other authors have reported the direct conversion of fibroblasts to neural progenitor cells [128] or tripotent neural precursor cells (iNSC) [129, 130] which have the advantage of being expandable.

The conversion of human fibroblasts from Alzheimer disease patients directly to functional neurons has been reported [131]. These reprogrammed neurons exhibit some of the hallmarks of the brain of the patients such as an altered processing of amyloid precursor protein and an increased production of Aβ.

Several works have pointed out that there are differences in gene expression patterns between iPS, embryonic stem cells, and somatic cells [132, 133]. These differences involve reprogramming process-dependent genes and those retained from somatic cells due to epigenetic memory. This fact should be taken into consideration to extrapolate the results obtained using these models. However, to better understand the relevance of these differences in the field of neurodegenerative diseases it would be necessary to compare the gene expression pattern of the reprogrammed cells with the somatic original cell as well as neuronal tissue from the same patient.

Although an increasing amount of work now uses either iPS, iN, or iNSC for modeling neurodegenerative diseases, in the case of Alzheimer, no mitochondrial function studies have been done so far in these models. Further efforts will be necessary to improve the efficiency of these protocols to increase neuron generation rate in order to perform this kind of approach.

Mitochondrial function is essential for neuronal differentiation and survival. Taking into consideration all the mitochondrial alterations described for Alzheimer disease, it is possible that the reprogramming of fibroblasts from AD patients into neurons could be more difficult. It has been described that mtDNA integrity is essential for mitochondrial maturation during differentiation of neuronal stem cells [134]; therefore, it is possible that lack of mtDNA integrity due to oxidative damage of nucleic acids may impair the reprogramming from fibroblasts to neurons. Also, a change in protein levels, like DLP1, previously mentioned, or other mitochondrial proteins such as prohibitin [135], could make AD fibroblasts more vulnerable to mitochondrial damage diminishing the efficiency of reprogramming. Therefore, the study of mitochondria during the reprogramming process might give important clues to understand not only the role of mitochondria during neuronal differentiation but also the relevance of AD-associated mitochondrial dysfunction in the neurodegeneration process.
